# Lovastatin as an Adjuvant to Lithium for Treating Manic Phase of Bipolar Disorder: A 4-Week, Randomized, Double-Blind, Placebo-Controlled Clinical Trial

**DOI:** 10.1155/2014/730505

**Published:** 2014-07-15

**Authors:** Ahmad Ghanizadeh, Motahhar OmraniSigaroodi, Ali Javadpour, Mohammad Hossein Dabbaghmanesh, Sara Shafiee

**Affiliations:** ^1^Research Center for Psychiatry and Behavioral Sciences, Shiraz University of Medical Sciences, Shiraz, Iran; ^2^Department of Psychiatry, School of Medicine, Shiraz University of Medical Sciences, Shiraz, Iran; ^3^Department of Neuroscience, School of Advanced Medical Sciences and Technologies, Shiraz University of Medical Sciences, Shiraz, Iran; ^4^Shiraz University of Medical Sciences, Shiraz, Iran; ^5^Shiraz Endocrine and Metabolism Research Center, Shiraz University of Medical Sciences, Shiraz, Iran

## Abstract

*Objectives*. Many patients with bipolar disorder suffer from metabolic disorder. Lovastatin is effective for treating major depression. This double-blind randomized placebo controlled clinical trial investigates whether lovastatin is a useful adjuvant to lithium for treating mania. *Methods*. Fifty-four patients with bipolar disorder-manic phase were randomly allocated into lovastatin or placebo group. The clinical symptoms were assessed at baseline, week 2, and week 4 using Young Mania Rating Scale. Adverse effects were checked. *Results*. Forty-six out of 54 patients completed this trial. The mania score in the lovastatin group decreased from 40.6 (11.1) at baseline to 12.9 (8.7) and 4.1 (5.4) at weeks 2 and 4, respectively. The score in the placebo group decreased from 41.0 (11.2) at baseline to 12.8 (8.07) and 5.8 (4.6) at weeks 2 and 4, respectively. However, there was no significant difference between groups at week 2 and week 4. The adverse effects rates were comparable between the two groups. No serious adverse effect was found. Tremor and nausea were the most common adverse effects. *Conclusions*. Lovastatin neither exacerbated nor decreased the symptoms of mania in patients with bipolar disorder. Current results support that the combination of lovastatin with lithium is tolerated well in bipolar disorder. The trial was registered with the Iranian Clinical Trials Registry (IRCT201302203930N18).

## 1. Introduction

Bipolar disorder is one of the most common inpatients psychiatric disorders. Physical activity of patients with bipolar disorder is lesser than nonusers of mental health services while their sedentary behavior is increased [1]. About 64.9% of them suffer from physical inactivity [2]. Furthermore, in some patients long-lasting adoption on unhealthy lifestyles may further make them prone to metabolic disorder [3].

In addition, most of patients with bipolar disorder and psychotic features should take antipsychotic medications that promote metabolic syndrome [4]. Moreover, adherence to treatment and pharmacotherapy is a major issue in patients with bipolar disorder. Cardiovascular disease is significantly associated with poor medication continuity [5].

The risk of diabetes mellitus, the metabolic syndrome [6], overweight, and dyslipidemia in patients with bipolar disorder is increased leading to the increased risk of cardiovascular disease and cerebrovascular disease. The rates of elevated fasting glucose (26.4%), diabetes (13.2%), hypertension (38.4%), hypertriglyceridemia (25.8%), low HDL-cholesterol (27.7%), general (38.4%), and abdominal obesity (59.1%) are marked [2]. All of these consequences lead to the increased rate of mortality [7]. Patients with bipolar disorder die 8.5 to 10 years earlier than the general population [8, 9]. Recently, this reduced life expectancy and its prevention have attracted the attention of researchers [9, 10].

Meanwhile, the primary prevention of these problems in psychiatry is neglected [7]. Therefore, it is recommended to use statins as a primary prevention for cardiovascular events, cerebrovascular events, and metabolic syndrome in patients with psychiatric disorders such as bipolar disorder [7].

On the other hand, lovastatin reduces the inflammation in animals [11]. Statins have neuroprotective effects [12] and reduce neuroinflammation [13, 14] and oxidative stress [14, 15]. Moreover, inflammation and oxidative stress are activity biological markers of bipolar disorder [16–18]. Bipolar disorder may be a “multisystemic inflammatory disease” [19].

Meanwhile, there is a controversy about the association of statin and depression. Lovastatin as an adjuvant treatment improves major depressive disorder [20]. Medical records of 46,249 patients aged 30–85 years showed that statin use was not associated with a higher risk of psychiatric disorders [21].

Statins are widely used for different purposes. For example, statins reduce the risk of prostate cancer [22]. However, statins caused ataxia in two patients with bipolar disorder [23].

Considering that lovastatin can be used as a preventive intervention for cardiovascular and cerebrovascular events and metabolic disorder in patients with bipolar disorder, its effects on depressive disorder [20], and the effect of statins on oxidative stress and inflammation, it is required to examine the effect of statins on manic phase of patients with bipolar disorder. Since lovastatin decreases depression, it might be hypothesized that lovastatin may exacerbate manic phase of bipolar disorder. Nevertheless, statins have anti-inflammatory and antioxidative stress effects. Therefore, it might be hypothesized that lovastatin may decrease manic symptoms in bipolar disorder. To the best of the authors' knowledge, no published trial has ever investigated the efficacy and safety of statins on bipolar disorder.

## 2. Methods

### 2.1. Design

This is a randomized, placebo-controlled, double-blinded clinical trial of lovastatin for treating manic phase of patients with bipolar disorder. This trial was conducted at the psychiatric hospital affiliated with Shiraz University of Medical Sciences from 2011 to 2013. Fifty patients were randomly allocated to either lithium (1–1.2 mEq/L) + lovastatin or lithium (1–1.2 mEq/L) + placebo in a 1 : 1 ratio. Random numbers were generated using a random number generator.

### 2.2. Medications

Placebo tablets similar to lovastatin tablets were provided by the Department of Pharmacology of Shiraz University of Medical Sciences. During this trial, the patients received one daily tablet of lovastatin or placebo in addition to their other prescribed medications. Lovastatin was administered as an adjuvant medication to the approved pharmacotherapy for bipolar mood disorder. Lovastatin was started with the dose of 10 mg/day and it was titrated up to 30 mg/day during one week. The patients received their normally prescribed medications during this trial. The patients who were taking medications before starting this trial continued them and no marked changes occurred. All the received medications will be reviewed.

### 2.3. Ethics

The trial was performed in accordance with principles of Good Clinical Practice and was approved by the Ethics Committee of Shiraz University of Medical Sciences. This trial is registered with the Iranian Clinical Trials Registry (http://www.irct.ir/) (IRCT201302203930N18). After providing written informed consent by the patients or their caregivers, whoever was applicable, the patients entered a 4-week trial.

### 2.4. Procedure

Patients from both genders aged more than 18 years with a primary* Diagnostic and Statistical Manual of Mental Disorders*, Fourth Edition, Text Revision (DSM-IV-TR) Axis I diagnosis of bipolar disorder were entered. Moreover, there was a score over than 19 on the Young Mania Rating Scale [24].

Exclusion criteria were pregnancy, breast feeding, those women in childbearing age who did not use contraceptive, regular use of lovastatin or other statins before entering this trial, taking diet pills or nutritional products, serious uncontrolled medical condition such as hypothyroidism or epilepsy, current substance dependency, alcohol abuse, clinically estimated mental retardation, cancer, and a history of renal or liver problems.

### 2.5. Clinical Assessments

Assessments occurred by a resident of psychiatry. Clinical assessments were performed three times including at baseline (week 0) and after 2 and 4 weeks of treatment onset. The patients were evaluated using the following measures.

#### 2.5.1. Clinical Global Impression Scale (CGI)

The change in Young Mania Rating Scale score during this trial was considered as the outcome measure of response.

#### 2.5.2. Adverse Effects

Adverse effects were systematically assessed using a checklist by a resident of psychiatry.

### 2.6. Statistical Analysis

A total of 34 patients (17 patients in each group) should be entered into this two-treatment parallel-design trial (power = 80 percent, two-sided 0.05 significance level, difference between treatments = 6.0, and standard deviation = 6).

Several *t*-tests were performed to examine the association of the mean years of age, weight (kilogram), and the mean numbers of daily smoking with the group. The association of gender, positive history of taking ECT, the rate of adverse effects, and the presence of psychotic features with the group was examined using Chi-Squared tests or Fisher's exact tests, whenever it was applicable. Repeated measure analysis of variance was used to examine the effect of treatment between the two groups.

## 3. Results

From 54 patients, there were 24 patients in the lovastatin group and 26 patients were in the placebo group. Four patients did not meet the YMRS score of 19 or higher.

**Figure 1:**
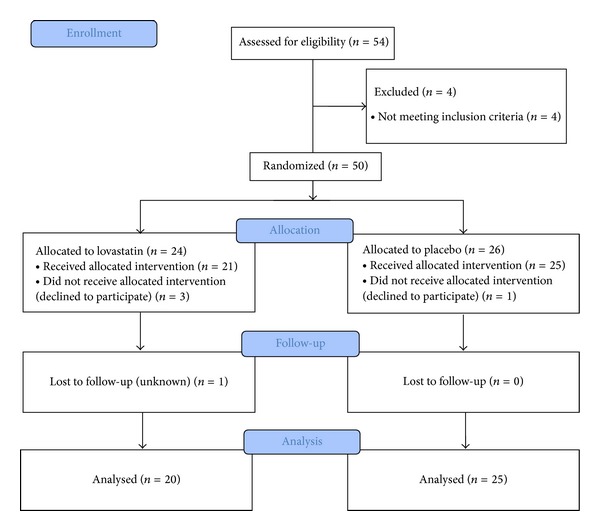
The flow chart of the patients.

Four patients withdrew their consent. Out of 54 patients, 45 patients completed 4 weeks of this trial (Figure 1).

At baseline, the lovastatin and placebo groups did not differ significantly on any of the gender ratio, age, mean of weight, positive history of taking ECT, current smoking, and positive current psychotic features (Table 1). Concurrent medications and their dosages are reported in Table 2. Ten (41.7%) of the patients in the lovastatin group and 11(42.3%) of the patients in the placebo group were taking lithium at the time of randomization (*χ* ^2^ = 0.002, *df* = 1, *P* = 0.9). The dose of lithium increased to have the serum lithium of 1.0 to 1.2 mEq/L.

### 3.1. Efficacy

**Figure 2:**
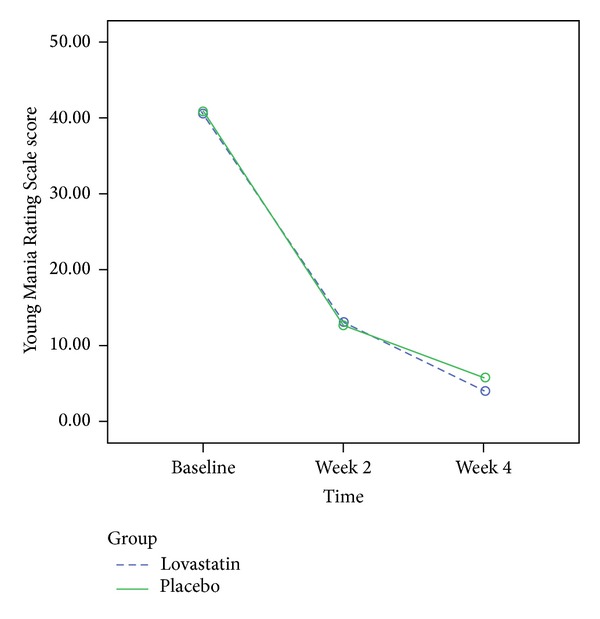
The changes of Young Mania Rating Scale score in the lovastatin and placebo group during this trial.

The YMRS score significantly decreased from baseline to week 2 and week 4 in both lovastatin and placebo groups (*F*(2,86) = 259.9, *P* < 0.0001). The score in the lovastatin group decreased from 40.6 (11.1) at baseline to 12.9 (8.7) and 4.1 (5.4) at weeks 2 and 4, respectively. The score in the placebo group decreased from 41.0 (11.2) at baseline to 12.8 (8.07) and 5.8 (4.6) at weeks 2 and 4, respectively. The interaction of time and group (time × group) was not statistically significant (*F*(2,86) = 0.15, *P* = 0.85). In addition, no between group difference was observed (*F*(1,43) = 510.0, *P* = 0.69). The lovastatin and placebo groups did not significantly differ regarding the YMRS score change from baseline to weeks 2 and 4 (Figure 2).

The CGI score decreased in both groups during this trial (*F*(2,62) = 6.42, *P* < 0.003). Nevertheless, groups were not associated with the CGI score at week 2 and week 4 (*F*(1,31) = 0.001, *P* = 1.0).

### 3.2. Safety and Tolerability

The rate of adverse effects was very low in both groups and no serious adverse effect was reported. The most common adverse effects were tremor and nausea. Both tremor and nausea were more common in placebo group than in lovastatin group. The entire checklist is displayed in Table 3.

## 4. Discussion

There is a controversy about the use of medications with antidepressant effects for treating bipolar disorder because it is proposed that antidepressants may induce a worsening of bipolar disorder [25]. This 4-week, double-blind, randomized controlled clinical trial of daily lithium plus lovastatin (30 mg/day) versus lithium plus placebo did not show statistically significant efficacy difference between the two groups. In both groups, the scores of YMRS significantly decreased during this trial.

In clinical practice, current results suggest that lovastatin could be administered for treating metabolic disorder, overweight, and dyslipidemia in patients with bipolar disorder- manic phase. Moreover, there is no serious concern about its negative effects on the course of bipolar disorder. Lovastatin not only treats their metabolic disorder but also may decrease the risk of cardiovascular problems leading to an increment in their quality of life. Current results do not support the hypothesis that antidepressive effects of lovastatin may exacerbate mania in bipolar disorder. Moreover, current results do not support our second hypothesis that lovastatin decreases mania symptoms due to its anti-inflammatory effects.

In addition, there was not any serious adverse effect found in this trial. No case with ataxia or myopathy was found. The most common adverse effect in both groups was tremor and nausea. Both of these adverse effects in the placebo group were more common than the lovastatin group. In fact, the patients tolerated this combination very well. Of course, it is possible that the duration of this trial was very short or the dose of lovastatin was not so high to allow the appearance of serious adverse effects such as rhabdomyolysis and myopathies [26]. However, a recent randomized controlled clinical trial showed that lovastatin was not effective for treating schizophrenia [27].

It would be optimal to include a large sample size with a long duration. However, our concern for possible exacerbation of mania by lovastatin and its adverse effect did not encourage us to include larger sample with more duration. Therefore, the generalization of the results for long term taking of lovastatin in bipolar disorder should be with caution. Moreover, lovastatin was administered as an adjuvant medication and the patients were allowed to take their other regular medications. The patients were with bipolar disorder type I. It is not clear whether the results could be generalized to other types of bipolar disorder. In addition, we assessed clinical symptoms. Further studies may investigate the effect of lovastatin with higher doses on some biological markers such as interleukins or inflammatory markers in patients with bipolar disorder.

In conclusion, the results of this first randomized double-blind, placebo-controlled clinical trial show that lovastatin as an adjuvant treatment to lithium neither exacerbates nor decreases mania symptoms in acute phase of mania in patients with bipolar disorder type I.

## Figures and Tables

**Table 1:** Basic characteristics of the patients in the two groups.

	Lovastatin group	Placebo group	Significance
Mean years of age (SD)	30.5 (8.1)	29.5 (10.8)	*t* = 0.38, *df* = 47, *P* = 0.7
Gender (number of male/female)	12/12	12/14	*χ* ^2^ = 0.07, *df* = 1, *P* = 0.78
Body Mass Index	25.6 (5.3)	25.8 (6.2)	*t* = 0.15, *df* = 43, *P* = 0.8
Positive history of taking ECT	0	2	—
Current psychotic feature	83.3%	100%	*χ* ^2^ = 4.71, *df* = 1, *P* = 0.03
Mean number of daily cigarette smoking	5 (18.6)	1.5 (6.0)	*t* = 0.88, *df* = 47, *P* = 0.3

**Table 2:** The rate and dose of concomitant medications by the groups.

Medication	Lovastatin group (number)	Placebo group (number)
Haloperidol (10 mg/day)	1	1
Perphenazine (24 mg/day)	4	2
Olanzapine (5–15 mg/day)	7	4
Risperidone (1–6 mg/day)	3	2
Thioridazine (100 mg/day)	1	1
Imipramine (25 mg/day)	1	0
Valproate sodium (200–800 mg/day)	6	3
Lamotrigine (100 mg/day)	0	1
Clonazepam (1.5 mg/day)	1	1
Propranolol (20 mg/day)	2	1
Bupropion	0	1
Clozapine (12.5–25 mg/day)	0	2
Carbamazepine (400 mg/day)	0	2

**Table 3:** The rate of adverse effects in both groups.

	Lovastatin group *N* (%)	Placebo group *N* (%)	*P* value
Constipation	0	1 (4.0%)	—
Dizziness	6 (26.1)	3 (33.3)	0.1
Hypotension	1 (4.3)	1 (4.0%)	—
Dry mouth	1 (4.3)	0	—
Blurred vision	0	0	—
Sweating	0	1 (4.0%)	—
Urinary disturbance	0	0	—
Tremor	10 (43.5)	18 (72)	0.04
Anorexia	0	0	—
Nervousness	3 (13)	9 (39.0)	—
Agitation	6 (30)	14 (56.0)	0.045
Headache	0	0	—
Insomnia	0	0	—
Diarrhea	0	0	—
Nausea	10 (43.5)	14 (56.0)	—
Drowsiness	6 (26.1)	3 (12.0)	—
Rash	0	2 (8.0)	—
Cramps	0	0	—
Myalgia	0	0	—
